# Allometric Optimization of Enrofloxacin Dosage in Growing Male Turkeys: Empirical Evidence for Improved Internal Exposure

**DOI:** 10.3390/antibiotics9120925

**Published:** 2020-12-18

**Authors:** Błażej Poźniak, Marta Tikhomirov, Karolina Motykiewicz-Pers, Kamila Bobrek, Marcin Świtała

**Affiliations:** 1 Department of Pharmacology and Toxicology, Faculty of Veterinary Medicine, Wroclaw University of Environmental and Life Sciences, ul. Norwida 31, 50-375 Wrocław, Poland; marta.tikhomirov@upwr.edu.pl (M.T.); karolina.motykiewicz-pers@upwr.edu.pl (K.M.-P.); marcin.switala@upwr.edu.pl (M.Ś.); 2 Department of Epizootiology and Clinic of Birds and Exotic Animals, Faculty of Veterinary Medicine, Wroclaw University of Environmental and Life Sciences, pl. Grunwaldzki 45, 50-366 Wrocław, Poland; kamila.bobrek@upwr.edu.pl

**Keywords:** allometry, dose optimization, fluoroquinolones, pharmacokinetics, turkeys

## Abstract

Rapid weight gain in turkeys causes a major change in the pharmacokinetics of drugs, leading to age-dependent variability in the internal exposure and, possibly, treatment failure and/or selection for antimicrobial resistance in young individuals. The aim of the study was to investigate whether a non-linear dosing protocol that accounts for the previously established allometric relation between enrofloxacin clearance and body weight (BW) may optimize the internal exposure to enrofloxacin in growing male turkeys. Enrofloxacin was administered four times, between the age of 5 and 16.5 weeks, when the turkeys’ BW increased from 1.47 to 14.92 kg. Enrofloxacin was given intravenously (i.v.) or orally at the dose calculated as follows: Dose = 30 × BW^0.59^. After i.v. administration, the internal exposure to the drug—quantified as the area under the concentration–time curve (AUC)—was showing little age-related variation. The coefficient of variation (CV) for AUC in all individuals (15.7%) was only slightly higher than within the age groups (5.4–13.7%). After oral drug administration, CV for AUC in all individuals (22.1%) was similar as within the age groups (8.7–32.2%). These results show that intra-species allometric scaling may be efficiently implemented in the non-linear approach to enrofloxacin dosage in turkeys in order to obtain a precise internal exposure for the optimal antimicrobial effect.

## 1. Introduction

As the veterinary use of antimicrobials in farm animals may contribute to the emergence of antimicrobial resistance, it is crucial to limit this use to precise strikes at precise dose, only when really needed [1]. To do that, we should understand the factors that drive the variability in the animals’ response to antimicrobial therapy. Some of these factors, like body weight gain in poultry, are highly predictable and, if understood and included into the optimized dosage regimens, may help to tailor the dose in order to eradicate the pathogen and prevent the spread of antimicrobial resistance in the often underdosed young individuals [2,3].

The selection of farm animal breeds characterized with higher productivity has a long history and has profoundly contributed to the impressive economic output of modern agriculture. Typical broiler chickens achieve a body weight of 3 kg within just 6 weeks of age, whereas heavy breeds of turkeys can reach a body weight of 15 kg within just 16 weeks. This rapid weight gain is achieved at significant physiological costs. The enormous gain in the mass and relative proportion of tissues is a significant challenge for the cardiovascular system and hemodynamics [4]. It was shown in both broiler chickens and turkeys that the relative cardiac output decreases significantly over a short time of intensive growth [5]. In clinical terms, this means that due to the high metabolic demands of expanding muscle tissue, a consistently lower proportion of blood is perfusing other organs, e.g., liver and kidney. This translates directly to a significant age-dependent decrease in the capability of these organs to metabolize and eliminate drugs administered to the fast-growing turkeys.

There is a body of evidence indicating that the pharmacokinetic (PK) processes that govern the disposition of numerous drugs undergo profound changes within just a few weeks of intensive growth in poultry. During this period, the clearance (CL) of metronidazole [5], florfenicol [6], amoxicillin [7], enrofloxacin [2], tylosin [8], doxycycline [9], or sodium salicylate [10] may decrease even three-fold and the resulting changes often far exceed the differences observed between different species. The implications of these changes may be profound as the same drug dosage (expressed in mg per kg body weight) will lead to very different effects in birds of different ages. In young individuals, there is a high risk of subtherapeutic concentrations, treatment failure, and selection of resistant bacterial strains. In contrast, the older individuals reaching the slaughter age may have unnecessary high concentrations, increasing the risk for drug residues and otherwise avoidable contamination of the environment with high amounts of drug in feces. Moreover, it may be associated with an unnecessary increase in the production costs. In our recent paper, we developed and validated an allometric model to predict enrofloxacin CL in growing turkeys administered intravenously at a standard dose of 10 mg/kg [2]. Based on this, we developed a novel non-linear dosage protocol in the form of a power function that reflects the change in the CL by taking the exponent value from the model: 0.59. The allometric coefficient was calculated based on the area under the curve (AUC) values determined for the treated turkeys and the final dosage protocol had the following formula:

Dose = 30 × (BW)^0.59^ (1)

where BW is the body weight. The precise explanation of how this dosing scheme was developed is provided in the material and methods section. According to this equation, in order to maintain constant AUC, turkeys weighing 1.5 kg should be administered a dose of 25.5 mg/kg, whereas those weighing 15 kg only 9.9 mg/kg of enrofloxacin.

The intra-species use of allometry has so far been limited to humans in order to optimize drug dosage for neonates [11]. In veterinary settings, allometric scaling is almost synonymous to the inter-species extrapolation of drug dosage from animals for which the dosage is well-established to species for which PK data is lacking [12]. It is, however, considered a rather imprecise tool as inter-species differences often outweigh the sole effect of the body weight [2,13]. Since the inter-species differences in drug metabolism and protein binding are eliminated in the intra-species allometric scaling, we hypothesize that this approach may also be very useful to increase the precision of antimicrobial usage in rapidly growing poultry breeds. This concept, however, has never been supported by empirical evidence in poultry so far and such evidence is needed if any practical conclusions for clinical application of drugs are to be drawn. Therefore, the aim of this study was to empirically validate this new concept of intra-species scaling of the dose in rapidly growing turkeys.

## 2. Results

**Figure 1:**
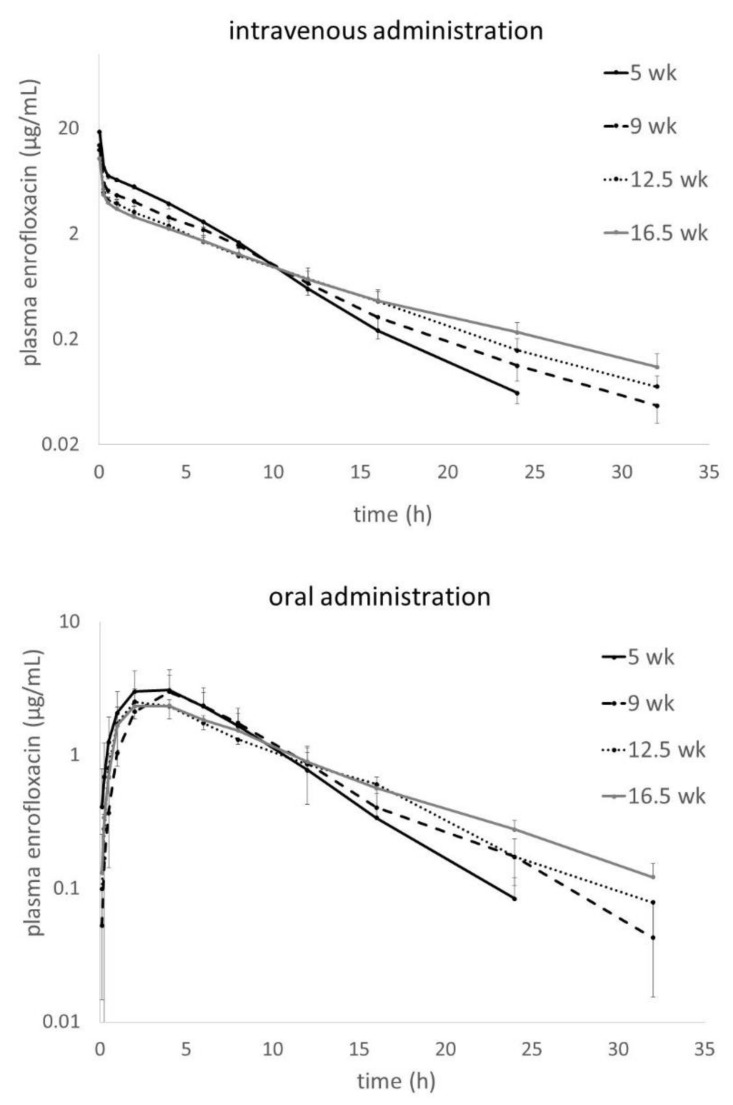
Plasma enrofloxacin concentrations after single intravenous (upper panel) and oral dose (lower panel) calculated according to the protocol: Dose = 30 × BW^0.59^.

**Figure 2:**
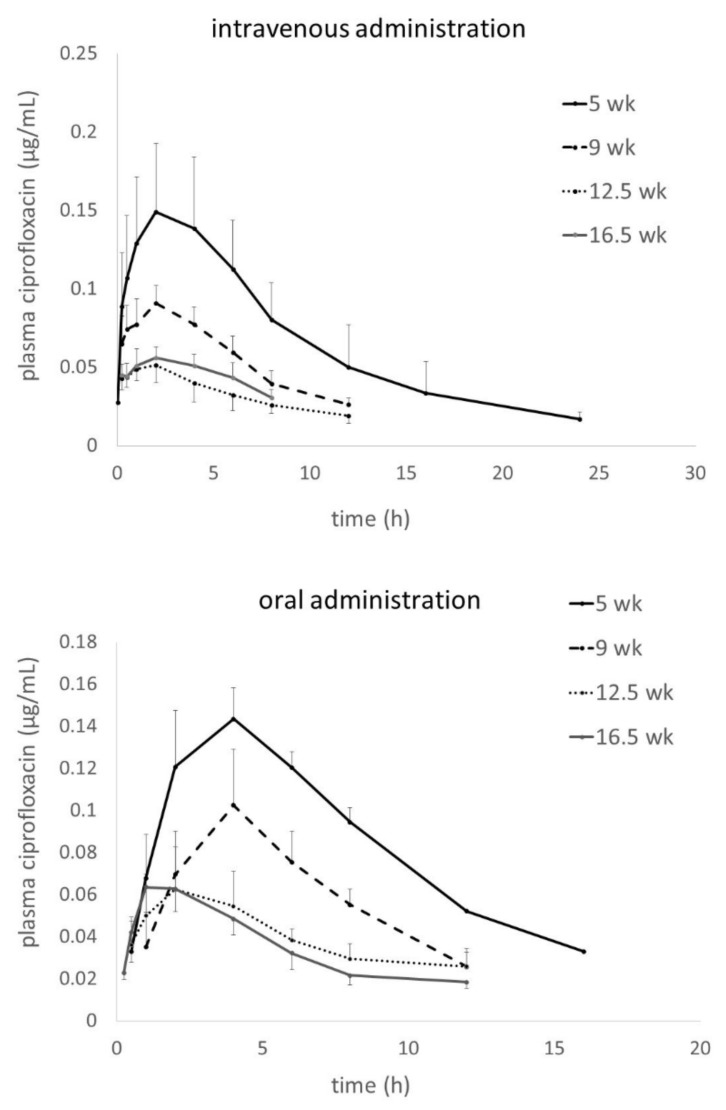
Plasma ciprofloxacin concentrations after single intravenous (upper panel) and oral dose (lower panel) of enrofloxacin calculated according to the protocol: Dose = 30 × BW^0.59^.

The curves for plasma enrofloxacin concentrations in turkeys at four different ages are shown in Figure 1. The upper panel presents the intravenous (i.v.) administration and the lower one the oral (p.o.) administration. For better visualization of age- and dose-dependent changes in profiles, the mean curves for the age groups were overlaid. For i.v. administration, after a very short distribution phase, a long clearly identifiable elimination phase is seen. Figure 2 shows plasma ciprofloxacin concentrations assessed in parallel to the enrofloxacin measurements shown in Figure 1. The differences between the age groups are clearly visible, with the youngest individuals showing the highest plasma ciprofloxacin concentrations.

Table 1 summarizes the PK parameters after i.v. administration. Although a minor age-dependent trend in AUCs and AUMC is seen, only the youngest group differs significantly from the other groups. The AUC_inf_ values are homogenous within the age groups as indicated by the CV of 5.4, 7.7, 13.7, and 11.0% in the 5-, 9-, 12.5-, and 16.5-week-old turkeys, respectively. When all the AUC_inf_ values are pooled together, the CV is only slightly higher: 15.7%. Although relatively small, the differences in AUC and AUMC translate to significant age-dependent prolongation of MRT and T_1/2el_, and are associated with less than a two-fold drop in CL. C_max_ is highest in the youngest turkeys but Vd_ss_ is very consistent in all age groups. The metabolic deethylation of enrofloxacin to ciprofloxacin (as reflected by the AUC_CIP_/AUC_ENR_ ratio) seems to be most efficient in the youngest turkeys. Although the efficacy of this process drops in older birds, the correlation with age in the last 7 weeks of the study seems to be less clear. In all age groups, the median T_max_ for ciprofloxacin was 2 h.

Table 2 shows the PK parameters for the oral administration. The differences between the AUC_inf_ are minor. The CV values within the age groups are less homogenous as compared to the i.v. study: 32.2, 21.3, 18.9, and 8.7% in the 5-, 9-, 12.5-, and 16.5-week-old turkeys, respectively. However, when all the AUC_inf_ values are pooled together, the CV is only 22%, indicating a minor effect of age on this parameter. On the other hand, AUMC and, as a result, MRT show an age-related increase similar to the i.v. study. This tendency is also seen in the T_1/2el_. The parameters describing absorption are less clear: MAT, T_max_, and C_max_ seem not to be affected by age. On the contrary, the F values seem to suggest that younger individuals absorb a smaller proportion of drug as compared to the older ones. The mean AUC_CIP_/AUC_ENR_ ratio for the oral administration is higher compared to the i.v. administration in all but the oldest group of turkeys. C_max_ is higher in the younger individuals and T_max_ seems to shorten with age. Individual enrofloxacin and ciprofloxacin measurements as well as individual pharmacokinetic parameters are provided in the Supplementary Materials (Spreadsheet S1 and S2, respectively).

## 3. Discussion

Allometric scaling is an analytical method, which assumes that physiological processes follow a power–law relationship with certain biological parameters, usually body weight [14,15]. In veterinary clinical settings, the application of allometric scaling is almost synonymous to inter-species scaling [12,16]. However, numerous scientific papers provide evidence that inter-species differences in physiology often outweigh the simple power relation of PK parameters to body weight and make allometric dose extrapolations risky [2,13]. These physiological differences may be explained by, e.g., various extent of liver metabolism, kidney structure, transporter expression, or protein binding [17]. Some authors suggest that the selection of species used in scaling to a specific group, e.g., birds or ruminants, may improve the predictive properties of such models [13,18]. However, the outcomes of these attempts are often still suboptimal [2,19,20]. Currently, these uncertainties limit the veterinary use of inter-species allometric scaling to a crude dose approximation tool for zoo veterinarians. However, for most drugs eliminated with urine, the power–law relationship between CL and body weight is a fact. Therefore, allometric scaling as a means to optimize drug dosage seems most useful in cases where physiological differences are minimal and the differences in the body weight are large [21]. Both these conditions are met in rapidly growing broilers and turkeys. In these animals, the most significant change developing over just a few weeks of life is the gradual decrease in the relative cardiac output [4,5], which is the driving force for the elimination of drugs excreted mainly by glomerular filtration [22]. Not surprisingly, this hemodynamic change follows the power–law relationship [6,15] and is responsible for the age-dependent shift in the PK of numerous drugs in broilers or turkeys [2,5,6,7,8,9]. Since this leads to the significant, even three-fold, difference in the internal exposure (AUC) between the animals of different age, we postulate the application of intra-species use of allometric scaling of dose in order to increase the precision of pharmacotherapy in broilers and turkeys. Why is uniform internal exposure so important? Fluoroquinolones are classified as concentration-dependent antimicrobials and their efficacy is best described by the ratio AUC/MIC (minimum inhibitory concentration for a pathogen) [23,24]. Ratios exceeding 100 are recommended for efficacy and prevention of resistance in infections caused by Gram-negative bacteria [23]. For Gram-positive bacteria, values of 30–55 may be satisfactory [25]. In our earlier study on enrofloxacin administered orally at a standard dose of 10 mg/kg, we found the AUC/MIC ratio of 22.6 and 58.2 in the 5- and the 16 week-old turkeys, respectively [2]. The assumed MIC breakpoint of 0.5 μg/mL was calculated for 235 field *E. coli* poultry isolates [26]. This age-dependent difference indicates a higher potential for therapeutic failure and resistance development in younger turkeys if treated at a standard dose. Moreover, it may explain the higher incidence of drug resistance found in *E. coli* isolates from young turkeys as compared to adult ones [3]. More recent studies suggest the use of the free fraction of the drug to calculate AUC/MIC [27]. Considering enrofloxacin protein binding in turkeys (30–40%) [2], the cited AUC/MIC ratios should be decreased by even 30–40% to obtain the actual values.

In the current study, we assessed the age-related variability in the internal exposure to enrofloxacin in turkeys after allometrically scaled drug dosage that accounts for the age-related change in CL. In the i.v. experiment, the CV for the values of AUC pooled from all age groups (15.7%) was only slightly higher compared to the CV values within the age groups (5.4–13.7%, which should be considered as the residual variability due to interindividual differences and assay error). In our previous study in which enrofloxacin was administered i.v. according to the standard dosage of 10 mg/kg, the CV value for the pooled AUC was 36% as compared to the within-group values of 5.8–9.6% [2]. This more than two-fold decrease in variability clearly indicates that the new dosage protocol managed to minimize the effect of age as a source of variability for the i.v. administration. However, it should be noted that the AUC values in the youngest individuals are significantly higher than in all other age groups, which suggests that, considering the need for the homogenous AUC, the new dosage protocol “overdoses” the drug in the youngest individuals. Since the new dose administered to the youngest turkeys was 25 mg/kg vs. the regular dose of 10 mg/kg, a possibility of saturated elimination should be considered as the underlying mechanism for the more-than-expected increase in the AUC [16]. The comparison of the T_1/2el_ in the current study (3.29 ± 0.16 h) with the value calculated for the regular 10 mg/kg dose in turkeys of that age (2.65 ± 0.24 h, [2]) indeed suggests that drug elimination was less efficient at the higher dosage. Additionally, CL was lower in turkeys treated at a higher dose: 0.59 ± 0.03 L/h/kg vs. 0.76 ± 0.07 L/h/kg in turkeys administered the dose of 10 mg/kg [2]. This “overdose” seems to be caused by the exponent used in our protocol: 0.59—value based on the actual age-dependent change in enrofloxacin CL in turkeys [2]. The deviation from the typical exponent value of 0.75 for the allometric model describing the relation between CL and BW seems to be related to the rapid weight gain in meat poultry, which is not typically observed in nature. The volume of the muscle tissue is expanding faster than the cardiovascular system so the relative blood flow to the clearing organs decreases. This leads to the faster than typical decrease in the relative CL with the increasing BW as reflected by the deeper inclination of the curve and, numerically, a lower exponent value. Although the currently used dosing protocol was developed based on an allometric model for CL of a specific drug in a specific species, it seems likely that the application of the commonly assumed exponent of 0.75 would result in a lack of such an overdose. Since the choice of the exponent also affects the calculation of the coefficient (see the material and method section), the revised dosage protocol would be as follows: D = 19.6 × BW^0.75^. In this case, the dose for a 1.5-kg turkey would be 17.7 mg/kg instead of 25.5 mg/kg, leading to a lower AUC. Although it has to be kept in mind that the exponent of 0.75 is an arbitrary choice [28], perhaps its use in drug dosage protocols for turkeys might be generalized to other concentration-dependent and renally excreted drugs without laborious drug-specific studies and still lead to satisfactory optimization of internal exposure. Possible candidates for dose optimization based on non-linear dosage may include other quinolones but also aminoglycosides or aminocyclitoles. Time-dependent drugs and those eliminated with bile seem to be less promising candidates for this approach. Validity of these hypotheses needs to be supported by experimental studies.

For oral administration, the age-dependent variability was similarly low. However, the basal interindividual variability within the age groups was significantly higher compared to the i.v. study due to the presence of the absorption phase. The CV for the pooled AUC values was only 22%, whereas the CV for the within-group values ranged from 8.7% up to even 32.2% (in the youngest individuals). In the previous study on the oral administration of enrofloxacin at the standard dose of 10 mg/kg, the effect of age was very clear, with the CV for the pooled AUC values 36% and the within-group CV values ranging from 11.8% to 12.6% [2]. This decrease in internal exposure variability by 14% (22% vs. 36%) indicates that the dosage protocol based on the allometric approach is also very efficient in oral enrofloxacin use in turkeys. However, since the presence of the absorption phase introduces another source of variability, the effects of dosage adjustment seem to be less pronounced compared to the i.v. administration. Interestingly, no apparent “overdose” similar to the i.v. administration was seen in the youngest turkeys and the AUC values did not differ significantly between the age groups. However, despite the overall decreased variability in the pooled AUC, the interindividual variability in the youngest group was very high (32.2%). This could be a result of variable absorption due to less predictable emptying of the crop in young turkeys [7,29]. It is also known that in young chickens, the intestinal expression of glycoprotein P is higher as compared to older individuals, which may complicate simple first-order absorption [30]. However, our earlier PK study on enrofloxacin orally administered to similar turkeys at a dose of 10 mg/kg showed a CV of only 12.4% [2]. Therefore, it seems to be more likely that this variability is caused mainly by a high dose of the drug resulting in limited, variable, or saturated absorption. Nevertheless, other parameters of absorption like F, C_max_, T_max_, and MAT, as well as the shape of the curve, do not support the hypothesis of saturated absorption. Regurgitation and resulting loss of drug are unlikely as the animals were closely monitored. Therefore, no single reason for this variability was identified. Importantly, the median AUC/MIC ratios in the age groups calculated for the MIC of 0.5 μg/mL [26] were between 51 and 64, suggesting an improvement in the expected clinical response as compared to the regular dosage of 10 mg/kg [2]. It should be kept in mind that the AUC/MIC value is strongly dependent on the MIC used in the calculation. If a breakpoint value of 0.25 [31] or 0.125 μg/mL is assumed [32], the AUC/MIC range in this study would be 104–128 or 204–256, respectively. Therefore, the current dosage protocol would provide the best outcome in infections caused by *E. coli* with a MIC ≤ 0.25 μg/mL, irrespective of the turkey’s age. This independence from the animal’s age is the fundamental advantage of the proposed dosing schedule because the classical dosage (10 mg/kg) would probably work only with the older turkeys but not with the young ones, leading to a high chance for the selection of resistant bacteria and treatment failure.

In contrast to enrofloxacin, ciprofloxacin concentration profiles look very different in respective age groups (Figure 2). As indicated by the metabolite/parent AUC ratio, the youngest turkeys were the most active metabolizers, which is in accordance with our previous findings [2] and may be related to the higher proportion of cardiac output reaching the liver as compared to the older turkeys [5]. This, together with the much higher enrofloxacin dose available for metabolism, contributed to relatively high ciprofloxacin concentrations in the youngest birds. However, accumulation of ciprofloxacin as a drug residue seems to be unlikely due to the generally low production rate of this metabolite and the expected higher elimination rate for ciprofloxacin as compared to enrofloxacin. Such a difference was confirmed in chickens [33] and seems to be a common trend also in other species [34]. When considering the overall antimicrobial efficacy, some authors suggest summing the concentrations of enrofloxacin and ciprofloxacin as the latter is pharmacologically active [35]. In turkeys, however, the low rate of ciprofloxacin production suggests that this practice would be of little clinical relevance.

We believe that the current study together with our earlier works provide enough evidence to abandon the popular paradigm in veterinary medicine: “one species—one dosage”. Considering the role of veterinary antimicrobial use in the emergence of antimicrobial resistance and the fact that due to economical and welfare conditions it seems impossible to completely eliminate the use of antimicrobials in food-producing animals, we need to explore every possible solution to limit the use of these drugs to precise strikes carried out only if needed and only at the effective dose. The selection of resistant bacteria is often caused by the exposure to subtherapeutic concentrations of antibiotics [36]. Therefore, it seems possible that the often observed high incidence of resistant microbes in young turkeys [3] is caused by insufficient drug concentrations obtained by standard drug dosage that does not account for age-dependent change in clearance. It seems very likely that the application of intra-species allometric scaling of antimicrobials would amend this problem. As chickens and turkeys are typically kept in large flocks of birds of the same age, and the drugs are administered mainly in drinking water, we believe that the application of this non-linear but still simple dosage protocol on a farm should be relatively easy. What may cause concerns is the significantly higher amount of drug that would be used in younger turkeys and, thus, the higher overall drug consumption on the farm. However, in the longer run and together with good biosecurity practices, the non-linear approach will give better pathogen eradication in younger individuals as compared to the current linear dosage. This may allow antimicrobial usage to be limited to precise and less frequent strikes only when the drug is really needed. Therefore, despite the individual doses being higher, they will be needed less frequently, and the overall drug consumption may not increase at all. Other possible difficulties may arise from the legal framework of drug registration, which binds the withdrawal times with precise drug dosage for a given formulation. Since birds reaching the slaughter age would have exactly the same dosage as before (approximately 10 mg/kg), the new approach should not increase the risk of residues. The application of intra-species scaling to drug dosage in poultry would require some flexibility from the regulatory sector. However, since it may bring some concepts of precision medicine to farms, we strongly believe it is worth it.

## 4. Materials and Methods

### 4.1. Animals

Twenty male turkeys (3-week-old, line BUT-9) were obtained from a commercial breeding facility in Poland. Turkeys were kept on straw bedding in an animal house with an ambient temperature of 20–23 °C and relative humidity of 50–60%. Water and commercial drug-free feed were provided *ad libitum*. Before the experiment started, two weeks were allowed for acclimatization. The birds were individually marked and randomly divided by lottery into two groups (*n* = 10 each): one for an i.v. and the other for the oral route of administration. The experiment was approved by the Local Animal Experimentation Committee in Wrocław, Poland (permit number 33/2016). All efforts were made to minimize animals’ suffering and to reduce the number of animals used. All procedures involving animals were performed in accordance with national and international laws and policies [37]. When the experiments were completed, the animals were examined by a veterinarian supervising animal welfare at the unit. After their satisfactory health condition was confirmed, turkeys were adopted by a private farmer and lost the status of experimental animals. After a few weeks, they were slaughtered in a routine way and used as dog food.

### 4.2. Pharmacokinetic Study

All turkeys were used in single-dose PK studies four times (parallel design with 4 phases and 2 groups differing by the route of administration, without blinding), that is, when the birds reached the age of 5, 9, 12.5, and 16.5 weeks. This age corresponded to the body weights of 1.47 ± 0.11, 4.62 ± 0.43, 9.16 ± 0.85, and 14.92 ± 1.22 kg, respectively. Before each experiment, the birds’ health was checked by physical examination. Only healthy individuals were included in the study (all animals remained healthy throughout the study). Enrofloxacin (Enrofloksacyna Vetos-Farma 50 mg/mL, injectable) was administered i.v. into vena brachialis or orally (Enrofloksacyna Vetos-Farma 100 mg/mL, oral solution) as a gavage into the crop at a single dose calculated based on the previously described dosing protocol: Dose = 30 × BW^0.59^. This protocol has been developed as follows: for allometric scaling, the CL may be interpreted as the proportionality factor between the dose (D) and the AUC (assuming first-order kinetics):

D = CL × AUC(2)

As the AUC is expected to be a constant value for the constant AUC/MIC, then dose becomes directly proportional to CL. In our recent study, we showed that the relation between CL and BW is not linear but follows the power law relation with the exponent of 0.59 [2]. Due to the aforementioned proportionality, the power law structure and the exponent for CL also applies to the dose:

D = cBW^0.59^ (3)

If the AUC for the heaviest turkeys obtained in the previous study [2] was considered satisfactory (to provide the highest AUC/MIC), then coefficient c is calculated as follows:

c = D/(BW^0.59^) (4)

by putting in the mean dose of ENR for the heaviest individuals (146 mg) and their mean body weight (14.6 kg). This leads to the following equation for ENR dosage in turkeys:

D = 30 BW^0.59^ (5)

This dosage protocol was expected to result in a constant AUC of around 37 mg × h/L (mean for the heaviest birds) in turkeys of all investigated age groups. In the current study, the dosage administered according to the equation above was 25.6, 16.0, 12.1, and 9.9 mg/kg in turkeys weighing 1.47, 4.62, 9.16, and 14.92 kg, respectively. All animals had been fasted for 10 h before drug administration and the experiments commenced at 7:00 A.M. The i.v. injection lasted 1 min. After that, blood samples (0.7 mL in the youngest birds, 1 mL in older turkeys) were collected from the jugular vein into heparinized syringes before the experiment as well as at 2, 15, and 30 min and 1, 2, 4, 6, 8, 12, 16, 24, and 32 h after drug administration (to reduce blood loss in the youngest individuals, sampling at 32 h was skipped in this group). For the p.o. studies, the first blood sampling took place at 7 min, and since 15 min, all sampling times were identical as in the i.v. study. After centrifugation (10 min, 3000× *g*), plasma samples were stored at −70 °C until being assayed for the concentration of enrofloxacin and its metabolite ciprofloxacin.

### 4.3. Determination of Enrofloxacin and Ciprofloxacin in Plasma

Plasma concentrations of enrofloxacin and ciprofloxacin were measured by means of a validated high-performance liquid chromatography (HPLC) method with UV detection as described in detail elsewhere [2]. Waters Alliance HPLC system (Waters, Milford, MA, USA) equipped with a 2996 PDA detector and a XTerra C18 MS (5 μm) 150 × 4.6 mm column (Thermo Fisher Scientific, Waltham, MA, USA) attached to an appropriate guard column were used. Plasma drug concentrations were calculated based on calibration curves prepared for analytical standards (Sigma-Aldrich, St. Louis, MO, USA). The limit of quantification (LOQ) for enrofloxacin was 0.017 µg/mL, and the limit of detection (LOD) was 0.006 µg/mL. For ciprofloxacin, LOD was 0.010 µg/mL and LOQ was 0.031 µg/mL. Assay validation for enrofloxacin and ciprofloxacin indicated an intra-assay coefficient of variation (CV) of 6.70% and 7.39%, respectively (at 6.25 µg/mL). An inter-assay CV for enrofloxacin was 7.54% and for ciprofloxacin was 9.07%. The recovery rate of enrofloxacin and ciprofloxacin was 90.31 ± 4.62% and 80.44 ± 2.09%, respectively.

### 4.4. Pharmacokinetic Analysis

The PK parameters of enrofloxacin and its metabolite were calculated based on a non-compartmental approach (TP4.1 software, ThothPro, Gdańsk, Poland). All data points, including values below LOQ (0.4% for enrofloxacin, 20% for ciprofloxacin), were included in the analysis according to recent recommendations [38]. The area under the concentration–time curve from time 0 to the last sampling (AUC_last_) and to infinity (AUC_inf_), the area under first moment curve from time 0 to infinity (AUMC_inf_), mean residence time (MRT), body clearance (CL), apparent volume of distribution at steady state (Vd_ss_), elimination half-life (T_1/2el_), and the maximal concentration (C_max_) were determined. For the calculation of T_1/2el_, at least three last datapoints from the linear portion of the terminal slope were used. For the p.o. study, AUC_last_, AUC_inf_, AUMC_inf_, MRT, T_1/2el_, as well as the peak plasma concentration (C_max_) and the time when it was observed (T_max_) were assessed. Mean absorption time (MAT) after oral administration was calculated as follows: MAT = mean MRTp.o.—mean MRTi.v. The bioavailability (F) of orally administered drug was calculated as follows: F (%) = 100 × [(mean AUC_inf_p.o. × Dose i.v.)/(mean AUC_inf_i.v.× Dose p.o.)]. For ciprofloxacin, due to low and variable concentrations, only AUC from time 0 to the last measurement (AUC_last_), C_max_, and T_max_ were assessed. To quantify the extent of drug metabolism, the ratio of AUC_last_ for ciprofloxacin and enrofloxacin was determined.

### 4.5. Statistical Analysis of Pharmacokinetic Parameters

Power analysis was calculated before the study and indicated that a minimum sample size of 10 turkeys per group is needed to detect a gradual decrease in CL of 0.1 L/h/kg among 4 age groups (starting with the value of 0.8 L/h/kg [2]) considering an alpha value of 0.05, a power of 80%, and a standard deviation of 0.2 L/h/kg (Statistica 13.3, Tibco, Palo Alto, CA, USA). The distribution of the PK parameters was assessed by the Shapiro–Wilk test (Statistica 13.3, Tibco, Palo Alto, CA, USA). The majority of parameters were normally distributed, and they are presented as mean and standard deviation (±SD). Statistical significance of the differences was assessed by one-way ANOVA with the post-hoc Tukey test. Since T_max_ values were found to lack normal distribution, they are presented as median and range, and the differences were assessed by the Kruskal–Wallis analysis of variance followed by the median test. In all cases, differences with *p* < 0.05 were considered significant. To assess the sources of variability in the internal exposure, the CV was calculated as the standard deviation/mean for the AUC_inf_ within the age group (inter-individual variability, age independent) as well as for the pooled values for all the age groups (age-dependent variability).

## 5. Conclusions

It was experimentally shown that the information on age-dependent changes in the pharmacokinetics of enrofloxacin in turkeys may be efficiently implemented in the non-linear approach to drug dosage in this species. This new dosage protocol may allow for a uniform internal exposure as indicated by the reduced variability in the AUC values in turkeys of different age. As a result, an optimized antimicrobial effect is expected to be achieved. This dose optimization can be done using very simple computational techniques and may be easily implemented in practice to increase the precision of antimicrobial treatment.

## Figures and Tables

**Table 1:** Pharmacokinetic parameters after single intravenous enrofloxacin administration to turkeys dosed according to the protocol: Dose = 30 × BW^0.59^.

Parameter		Unit	Age Groups (Body Weight ± SD), *n* = 10 Each			
			5 Weeks (1.55 ± 0.07 kg)	9 Weeks (4.74 ± 0.46 kg)	12.5 Weeks (9.34 ± 1.05 kg)	16.5 Weeks (15.11 ± 1.61 kg)
ENR	AUC_inf_	mg × h/L	42.58 ± 2.30 ^a^	35.49 ± 2.73 ^b^	31.71 ± 4.34 ^b^	31.67 ± 3.48 ^b^
	CV_group_	%	5.4	7.7	13.7	11.0
	CV_pooled_	%	15.7			
	AUC_last_	mg × h/L	42.32 ± 2.29 ^a^	35.24 ± 2.69 ^b^	31.20 ± 4.29 ^b,c^	30.74 ± 3.14 ^c^
	AUMC_inf_	mg × h^2^/L	191.0 ± 14.4 ^a^	204.4 ± 27.6 ^a^	222.7 ± 42.2 ^a,b^	259.8 ± 51.3 ^b^
	MRT_inf_	h	4.48 ± 0.17 ^a^	5.74 ± 0.43 ^b^	6.99 ± 0.56 ^c^	8.14 ± 0.75 ^d^
	CL_(rel)_	L/h/kg	0.59 ± 0.03 ^a^	0.45 ± 0.05 ^b^	0.39 ± 0.05 ^c^	0.33 ± 0.04 ^d^
	Vd_ss(rel)_	L/kg	2.53 ± 0.13 ^a^	2.47 ± 0.17 ^b^	2.51 ± 0.27 ^b,c^	2.32 ± 0.18 ^c^
	T_1/2el_	h	3.29 ± 0.16 ^a^	4.52 ± 0.30 ^b^	5.50 ± 0.46 ^c^	6.38 ± 0.58 ^d^
	C_max_	µg/mL	18.56 ± 1.29 ^a^	13.90 ± 2.13 ^b^	12.48 ± 1.78 ^b,c^	10.43 ± 2.02 ^c^
CIP	AUC_last_	mg × h/L	1.49 ± 0.44 ^a^	0.64 ± 0.11 ^b^	0.36 ± 0.11 ^b^	0.36 ± 0.04 ^b^
	C_max_	µg/mL	0.15 ± 0.04 ^a^	0.09 ± 0.01 ^b^	0.05 ± 0.01 ^c^	0.06 ± 0.01 ^c^
	T_max_	h	2 (1–6)	2 (0.5–2)	2 (0.25–4)	2 (0.25–4)
AUC_CIP_/AUC_ENR_		%	3.51 ± 1.03 ^a^	1.84 ± 0.37 ^b^	1.15 ± 0.28 ^b^	1.18 ± 0.15 ^b^

Values expressed as mean ± SD (for T_max_ median and range). Abbreviations: BW—body weight, ENR—enrofloxacin, CIP—ciprofloxacin, AUC_inf_—area under the curve from time 0 to infinity, CV_group_—coefficient of variation of AUC_inf_ within the age group, CV_pooled_—coefficient of variation of AUC_inf_ for all age groups, AUC_last_—area under the curve from time 0 to the last quantifiable value, AUMC_inf_—area under the first moment curve, MRT—mean residence time, CL_(rel)_—relative body clearance, Vd_ss(rel)_—relative volume of distribution at steady state, T_1/2el_—elimination half-life, C_max_—maximal concentration, T_max_—time when the C_max_ for CIP was reached, AUC_CIP_/AUC_ENR_—the ratio of AUC_0-last_ for the metabolite and the parent compound. Values in a row not sharing a common superscript letter are statistically different, *p* < 0.05. Lack of superscript indicates lack of statistical difference.

**Table 2:** Pharmacokinetic parameters after single oral enrofloxacin administration to turkeys dosed according to the protocol: Dose = 30 × BW^0.59^.

Parameter		Unit	Age Groups (Body Weight ± SD), *n* = 10 Each			
			5 Weeks (1.45 ± 0.07 kg)	9 Weeks (4.53 ± 0.30 kg)	12.5 Weeks (8.98 ± 0.48 kg)	16.5 Weeks (14.53 ± 1.61 kg)
ENR	AUC_inf_	mg × h/L	28.38 ± 9.15	27.07 ± 5.12	27.82 ± 5.93	28.92 ± 2.52
	CV_group_	%	32.2	21.3	18.9	8.7
	CV_pooled_	%	22.1			
	AUC_last_	mg × h/L	27.90 ± 9.09	26.31 ± 5.57	27.52 ± 5.93	27.77 ± 2.36
	AUMC_inf_	mg × h^2^/L	194.7 ± 66.2 ^a^	256.6 ± 95.1 ^a,b^	232.7 ± 51.4 ^a,b^	300.5 ± 48.0 ^b^
	MRT_inf_	h	6.86 ± 1.27 ^a^	9.48 ± 3.14 ^b^	8.48 ± 1.45 ^a,b^	10.36 ± 1.23 ^b^
	MAT *	h	2.38	3.74	1.50	2.22
	T_1/2el_	h	3.76 ± 0.55 ^a^	5.76 ± 2.26 ^b,c^	4.49 ± 0.69 ^a,b^	6.61 ± 0.56 ^c^
	C_max_	µg/mL	3.19 ± 1.18	2.62 ± 0.45	3.13 ± 0.72	2.55 ± 0.39
	T_max_	h	3 (1–6)	2 (2–16)	4 (4–6)	2 (2–4)
	F *	%	66.7	76.3	87.8	91.29
CIP	AUC_last_	mg × h/L	1.36 ± 0.22 ^a^	0.67 ± 0.14 ^b^	0.44 ± 0.12 ^c^	0.38 ± 0.08 ^c^
	C_max_	µg/mL	0.15 ± 0.04 ^a^	0.11 ± 0.02 ^b^	0.06 ± 0.02 ^c^	0.07 ± 0.01 ^c^
	T_max_	h	4 (2–8) ^a^	4 (4–6) ^a^	2 (0.5–16) ^a,b^	1.5 (0.5–2) ^b^
AUC_CIP_/AUC_ENR_		%	6.01 ± 3.63 ^a^	2.64 ± 0.60 ^b^	1.76 ± 0.94 ^b^	1.37 ± 0.27 ^b^

Values expressed as mean ± SD (for T_max_ median and range). Abbreviations: BW—body weight, ENR—enrofloxacin, CIP—ciprofloxacin, AUC_inf_—area under the curve from time 0 to infinity, CV_group_—coefficient of variation of AUC_inf_ within the age group, CV_pooled_—coefficient of variation of AUC_inf_ for all age groups, AUC_last_—area under the curve from time 0 to the last quantifiable value, AUMC_inf_—area under the first moment curve, MRT—mean residence time, MAT—mean absorption time, T_1/2el—_elimination half-life, C_max_—maximal concentration, T_max_—time when the C_max_ is reached, F—bioavailability, AUC_CIP_/AUC_ENR_—the ratio of AUC_last_ for the metabolite and the parent compound. * Calculated for the mean value from the i.v. administration and the mean value from oral administration. Values in a row not sharing a common superscript letter are statistically different, *p* < 0.05. Lack of superscript indicates lack of statistical difference (not valid for F and MAT as statistically incomparable).

## Supplementary Materials

The following are available online at https://www.mdpi.com/2079-6382/9/12/925/s1. Spreadsheet S1: Individual enrofloxacin and ciprofloxacin measurements, Spreadsheet S2: Individual pharmacokinetic parameters.
